# Supplementary material for: Deciphering Genetic Alterations of Taiwanese Patients with Pancreatic Adenocarcinoma through Targeted Sequencing
Source: Int J Mol Sci. 2022 Jan 29;23(3):1579. doi: 10.3390/ijms23031579 (PMC8835797; doi:10.3390/ijms23031579)
Supplement: Supplementary file 1 [file ijms-23-01579-s001.zip › ijms-1562352-SI/Supplementary materials.pdf]

Supplementary materials

Legends for Supplementary Figures

Supplementary Figure S1. Variant impact heat map of five Taiwanese PAC samples assayed with the OCP. Variant impacts were color-coded using the associated score values. Default scores are unknown: 0, synonymous: 1, missense: 2, non-frameshift block substitution: 3, non-frameshift insertion: 4, non-frameshift deletion: 4, nonsense: 5, stop-loss: 6, frameshift block substitution: 7, frameshift insertion: 7, frameshift deletion: 7, and splice variant: 8 (PAC: pancreatic adenocarcinoma, OCP: Oncomine Comprehensive Panel).

Supplementary Figure S2. Variant impact heat map of mucinous PAC (left) and PAC with pancreatic intraepithelial neoplasm precursor (right) samples assayed with the CHP. Variant impacts were color-coded using the associated score values. Default scores are unknown: 0, synonymous: 1, missense: 2, non-frameshift block substitution: 3, non-frameshift insertion: 4, non-frameshift deletion: 4, nonsense: 5, stop-loss: 6, frameshift block substitution: 7, frameshift insertion: 7, frameshift deletion: 7, and splice variant: 8 (PAC: pancreatic adenocarcinoma, Mucin: PAC with mucinous precursor, PIN: PAC with pancreatic intraepithelial neoplasm precursor, CHP: Cancer Hotspot Panel).

Supplementary Figure S3. OncoPrinter of 176 TCGA PAC samples with genes ordered as in Figure 3 (TCGA: The Cancer Genome Atlas, PAC: pancreatic adenocarcinoma).

Supplementary Figure S4. OncoPrinter of 176 TCGA PAC samples with genes ordered as in Figure 4; germline mutations and alterations of unknown significance are excluded (TCGA: The Cancer Genome Atlas, PAC: pancreatic adenocarcinoma).

Supplementary Tables

Supplementary Table S1. PAC samples (X-axis) assayed by the CHP tabulates with mutant genes (Y-axis), with (top) and without (bottom) weighted by the number of called variants (PAC: pancreatic adenocarcinoma, CHP: Cancer Hotspot Panel).

	P1	P13	P14	P15	P18	P20	P22	P27	P29	P32	P33	P35	P39	P40	P41	P43	P44	P45	P46	P48	P50	P53	P55	P56	P57	P58	P6	P60	P64	P65	P66	P68	P8	P9
CDKN2A																						1								1				
CTNNB1														1						1														
EGFR																					1		1											
GNAS						2									2																			
HRAS													1																					
IDH1																					1										1			
KIT			1										1								1		1										1	1
KRAS	1	1	1	1	1	1		1	1		1		1			1		1	1	1		1	1	1	1	1		1			1	1	1	
MET				1												1				1				1										
PDGFRA			1				1			1	1	1			1			1								1				1	1			
PTEN			1			1																			1							3		
SMARCB1	1						1		1						1								1										1	
SMO																																		1
STK11						1									1																			
TP53		2	2	5	2			4	4		1														2		1		1				5	

	P1	P13	P14	P15	P18	P20	P22	P27	P29	P32	P33	P35	P39	P40	P41	P43	P44	P45	P46	P48	P50	P53	P55	P56	P57	P58	P6	P60	P64	P65	P66	P68	P8	P9
CDKN2A																						1								1				
CTNNB1														1						1														
EGFR																					1		1											
GNAS						1									1																			
HRAS													1																					
IDH1																					1										1			
KIT			1										1								1		1										1	1
KRAS	1	1	1	1	1	1		1	1		1		1			1		1	1	1		1	1	1	1	1		1			1	1	1	
MET				1												1				1				1										
PDGFRA			1				1			1	1	1			1			1								1				1	1			
PTEN			1			1																			1							1		
SMARCB1	1						1		1						1								1										1	
SMO																																		1
STK11						1									1																			
TP53		1	1	1	1			1	1		1														1		1		1				1	

Supplementary Table S2. Recurrent COSMIC IDs from Taiwanese PAC assayed with the CHP (PAC: pancreatic adenocarcinoma, CHP: Cancer Hotspot Panel).

COSMIC ID	Called frequency	Gene	FATHMM* prediction
COSM22413	10	*PDGFRA*	Pathogenic (score: 0.88)
COSM520	10	*KRAS*	Pathogenic (score: 0.98)
COSM521	7	*KRAS*	Pathogenic (score: 0.98)
COSM1090	6	*SMARCB1*	
COSM28026	5	*KIT*	Pathogenic (score: 0.74)
COSM518	4	*KRAS*	Pathogenic (score: 0.98)
COSM710	4	*MET*	
COSM5915	3	*PTEN*	
COSM12473	2	*CDKN2A*	
COSM21360	2	*STK11*	
COSM27895	2	*GNAS*	
COSM94388	2	*GNAS*	
NOCOSMIC10	2	*IDH1*	

*FATHMM: functional analysis through hidden Markov models

Supplementary Table S3. Predicted recurrent pathogenic COSMIC IDs from Taiwanese PAC assayed with the CHP (PAC: pancreatic adenocarcinoma, CHP: Cancer Hotspot Panel).

Mutation ID	Gene Symbol	Amino acid mutation	CDS mutation
COSM22413	*PDGFRA*	p.V824= (Substitution - coding silent)	c.2472C>T (Substitution, position 2472, C➞T)
COSM520	*KRAS*	p.G12V (Substitution - Missense, position 12, G➞V)	c.35G>T (Substitution, position 35, G➞T)
COSM521	*KRAS*	p.G12D (Substitution - Missense, position 12, G➞D)	c.35G>A (Substitution, position 35, G➞A)
COSM518	*KRAS*	p.G12R (Substitution - Missense, position 12, G➞R)	c.34G>C (Substitution, position 34, G➞C)

Supplementary Table S4. Clinical and histological features of Taiwanese PAC patients assayed with the CHP (PAC: pancreatic adenocarcinoma, CHP Cancer Hotspot Panel).

Sample	Sex	Histology	Grade	LVI*	PNI*	Pre-cancerous lesion (if any)	Stage	Mucin
P48	F	Ductal	1	LVI	PNI	PIN3	pT3N1M1	mucin intensive
P50	M	Ductal	2	LVI	PNI	PIN2	pT3N1	mucin irregular
P53	F	Ductal	2	LVI	PNI	PIN3	pT3N1	mucin irregular
P55	M	Ductal	2	LVI	PNI	PIN3	pT3N0	mucin irregular
P56	M	Ductal	2	LVI	PNI	PIN3	pT3N1	mucin irregular
P57	F	Ductal	1	LVI	PNI	PIN3	pT3N1	mucin intensive
P58	F	Ductal	2	LVI	PNI	PIN3	pT3N1	mucin irregular
P60	M	Ductal	2	LVI	PNI	PIN3	pT3N0	mucin irregular
P64	M	Ductal	2	LVI	PNI		pT3N1	
P65	M	Ductal	3	LVI	PNI	PIN	pT3N1M1	mucin abortive
P66	F	Ductal	2	LVI	PNI	PIN3	pT3N1	mucin irregular
P68	M	Ductal	2	LVI	PNI	PIN3	pT3N1	mucin irregular
P01	F	Ductal	3	LVI	no		pT3N1	

P06	F	Ductal	1	LVI	PNI	PIN3	pT3N1	
P08	M	Ductal	3	LVI	PNI		pT3N1	
P09	M	Ductal	2	LVI	PNI		pT3N1	
P11	F	Ductal						
P13	F	Ductal	3	LVI	PNI		pT3N0	
P14	M	Ductal	2	LVI	PNI		pT3N1	
P15	F	Ductal	3	LVI	PNI	IPMN	pT3N1	
P18	M	Ductal	3	LVI	PNI		pT3N0	mucin focally positive
P20	M	Ductal	2	LVI	PNI		pT3N1	mucin irregular
P22	M	Ductal	2	LVI	PNI		pT3N0	mucin irregular
P24	M	Invasive	1	LVI	PNI	Mucinous cystic neoplasm	pT3N0	
P27	F	Ductal	2	LVI	PNI		pT3N1	mucin irregular
P29	M	Ductal	2	LVI	PNI		pT3N1	
P32	F	Ductal	2	LVI	PNI		pT3N1	mucin irregular
P33	M	Ductal	3	LVI	PNI	IS	pT3N1	mucin irregular
P35	F	Ductal	3	LVI	PNI		pT3N1	

P37	F	Ductal	3	LVI	PNI	PIN	pT3N1	mucin abortive
P38	M	Colloid	2	LVI	PNI		pT3N1	mucin marked
P39	M	Ductal	3	LVI	PNI		pT3N0	
P40	M	Ductal	3	LVI	PNI		pT3N1	
P41	M	Ductal	2	LVI	PNI	PIN3	pT3N1	
P43	F	Ductal	2	LVI	PNI	PIN	pT3N1	
P44	M	Undifferentiated+ductal	4	LVI	PNI	PIN3	pT3N1M1	
P45	M	Ductal	2	LVI	PNI		pT3N1	
P46	M	Ductal	2	LVI	PNI		pT3N1	mucin occasional

(*LVI: lympho-vascular invasion, PNI: peri-neural invasion)
