# Supplementary figures and images for: Deciphering Genetic Alterations of Taiwanese Patients with Pancreatic Adenocarcinoma through Targeted Sequencing
Source: Int J Mol Sci. 2022 Jan 29;23(3):1579. doi: 10.3390/ijms23031579 (PMC8835797; doi:10.3390/ijms23031579)

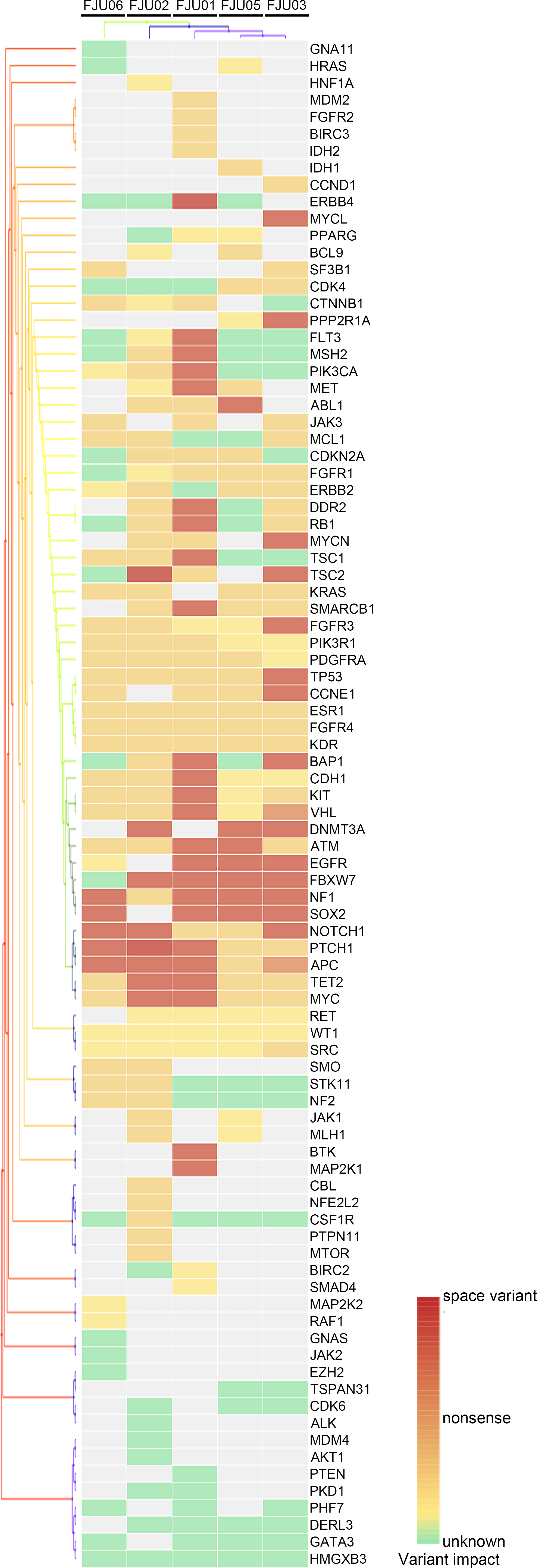

Supplement: Supplementary file 1 [file ijms-23-01579-s001.zip › ijms-1562352-SI/Fig S1.tiff]

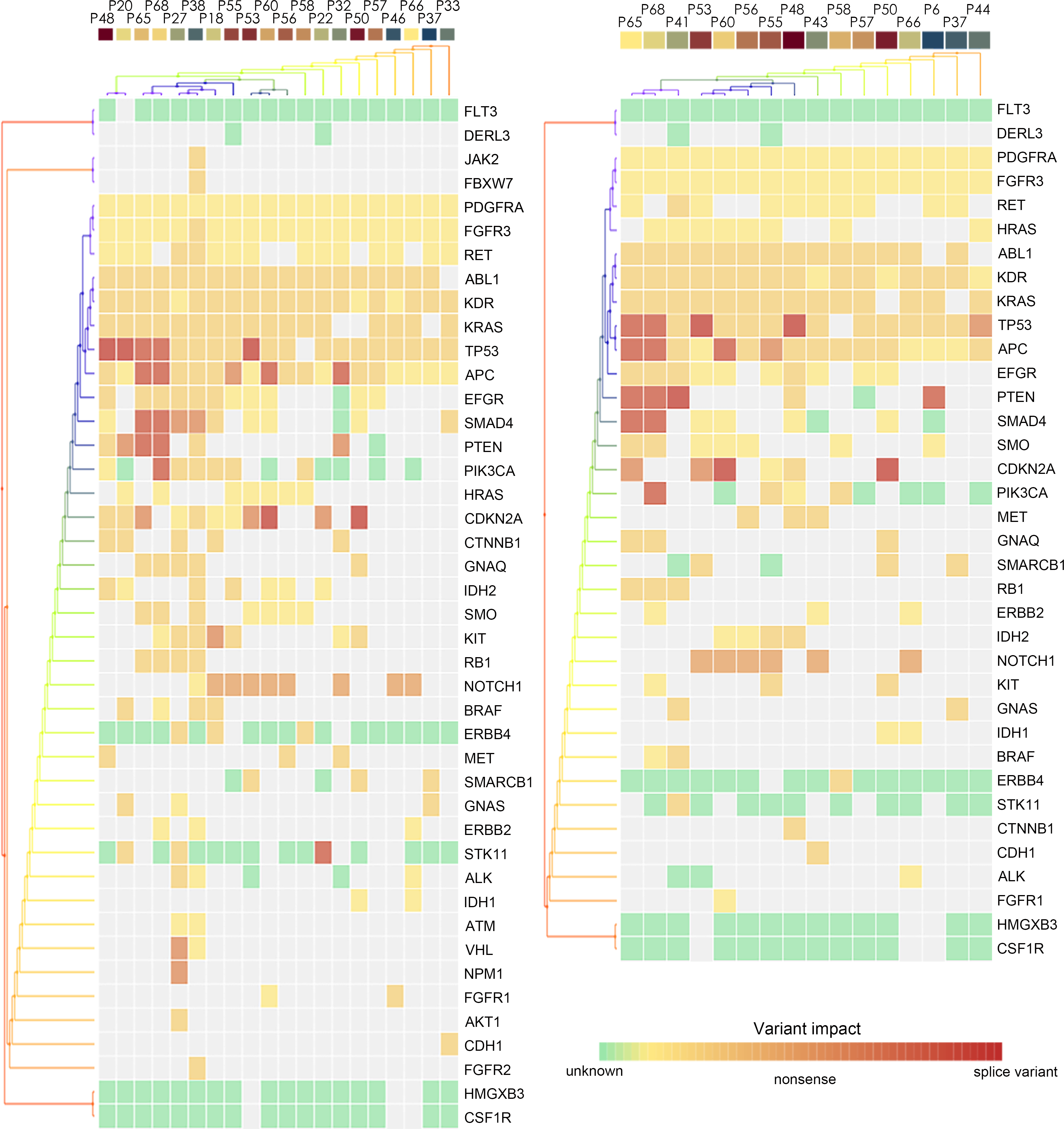

Supplement: Supplementary file 1 [file ijms-23-01579-s001.zip › ijms-1562352-SI/Fig S2.tiff]
